# Fear of Cancer Progression: A Comparison between the Fear of Progression Questionnaire (FoP-Q-12) and the Concerns about Recurrence Questionnaire (CARQ-4)

**DOI:** 10.3390/healthcare12040435

**Published:** 2024-02-08

**Authors:** Andreas Hinz, Thomas Schulte, Anja Mehnert-Theuerkauf, Diana Richter, Annekathrin Sender, Hannah Brock, Michael Friedrich, Susanne Briest

**Affiliations:** 1 Department of Medical Psychology and Medical Sociology, Comprehensive Cancer Center Central Germany, University Medical Center Leipzig, 04103 Leipzig, Germany; 2 Rehabilitation Clinic Bad Oexen, 32549 Bad Oeynhausen, Germany; 3 Department of Gynecology, Comprehensive Cancer Center Central Germany, University Medical Center Leipzig, 04103 Leipzig, Germany

**Keywords:** fear of progression, fear of cancer recurrence, health anxiety, psycho-oncology, cancer, psychometrics

## Abstract

As cancer patients often suffer from fear of cancer progression (FoP), valid screening for FoP is of high relevance. The aims of this study were to test psychometric properties of two FoP questionnaires, to determine their relationship to other anxiety-related constructs, and to analyze the impact of sociodemographic and clinical factors on the FoP. Our sample consisted of *n* = 1733 patients with mixed cancer diagnoses. For measuring FoP, the Fear of Progression questionnaire (FoP-Q-12) and the Concerns About Cancer Recurrence Questionnaire (CARQ-4) were used. The mean scores of the FoP-Q-12 and the CARQ-4 were 30.0 ± 10.4 and 16.1 ± 10.8, respectively, indicating relatively high levels of FoP. Both questionnaires showed excellent internal consistency coefficients, α = 0.895 and α = 0.915, respectively. The correlation between the two FoP questionnaires was *r* = 0.72. Female patients reported more FoP than male patients (*d* = 0.84 and *d* = 0.54, respectively). There was a nonlinear age dependency of FoP, with an increase found in the age range from 18 to 50 years and a decrease in the older age range. Radiation, chemotherapy, and antibody therapy, but not surgery, lead to an increase in FoP. Both questionnaires show good psychometric properties and can be recommended for use in an oncological routine. Female patients and patients in the middle-age range deserve special attention from healthcare providers.

## 1. Introduction

Fear of cancer progression (FoP) is a psychological burden frequently experienced by cancer patients [1,2,3]. Despite improvements in the early detection and treatment of cancer, many patients and survivors are confronted with the possibility that their cancer will progress or return [4]. FoP can be understood as the fear of cancer recurring or progressing in the same organ or another organ. In a comprehensive review study, the prevalence of FoP was found to be between 0 and 86% [5]. This extremely large range indicates that the case definition of FoP is highly dependent on the instrument chosen and the cut-off values used. This also means that a comparison of different instruments to determine FoP is essential.

Though FoP can be understood as a normal response to a real risk [6], exaggerated levels of FoP can lead to functional impairment, clinical symptomatology, utilization of healthcare services, and problems with adherence to medical treatment [7].

FoP is associated with fatigue and reduced quality of life [8]; illness representation and problematic psychological adjustment [9]; coping strategies such as denial [10]; intrusive thoughts and intolerance of uncertainty [11]; low levels of self-efficacy [12], optimism [13], and social support [14]; and medical side effects [15].

FoP and fear of cancer recurrence (FCR) are very similar constructs [16], and the instruments for assessing FoP and FCR show great overlap. In the context of this article, we will not distinguish between these concepts, preferring to use the term FoP for both constructs.

FoP is associated with health worries, depression, low levels of mental health, and, in particular, with generalized anxiety. For example, the correlations between FoP instruments and HADS anxiety were 0.68 [17] and 0.65 [18]. It has not yet been tested whether correlations between questionnaires that claim to measure FoP are stronger than those with scales of generalized anxiety or related constructs.

There are sex and age differences in the FoP. Females generally report higher levels of FoP than males [3,19], which reflects the generally higher mean levels of anxiety in women, both in patient groups as well as in the general population [20].

Concerning age differences, the results are mixed. While some studies found higher FoP mean scores for younger patients [3,15,19,21], another study reported an increase in FoP with increasing age [22], and further studies observed no significant age differences [18,23,24]. A meta-analysis comprising data of 46 single studies reported a negative association between FoP and age (beta = −0.10) for cancer patients [3]. The seemingly contradictory results may be explained by a non-linear relationship between age and FoP, which is only insufficiently represented by a linear correlation or regression coefficient. Therefore, there is a need for further research into sex and age differences in FoP.

Several studies on FoP included different types of cancer and compared the respective FoP mean scores of the diagnosis groups. A general finding was that patients with breast cancer, cancer of the female genital organs, and lung cancer reached relatively high FoP scores, while patients with testicular cancer or prostate cancer showed low scores [3,19]. When comparing the tumor types with regard to FoP, however, it should be noted that tumor type and gender are partially confounded; therefore, it remains unclear to what extent the higher values for breast cancer and gynecological cancer in patients are due to the female gender.

Precise detection and surveillance of cancer patients with high levels of FoP is important for preventing or reducing negative consequences of exaggerated FoP. Therefore, several instruments have been developed to assess FoP in cancer [25], ranging from ultra-short 1-item instruments [26,27] to the 43-item Fear of Progression Questionnaire (FoP-Q) [28]. A frequently used and validated instrument for assessing FoP in cancer patients is a short form of the FoP-Q, the FoP-Q-12 [29]. A recent study further condensed this questionnaire and extracted five items, arriving at the 5-item Fear of Progression Questionnaire–Rapid Screener (FoP-Q-RS) [30]. Another recently developed short questionnaire for measuring FoP is the Concerns About Recurrence Questionnaire CARQ-4 [31]. This instrument includes a question that asks about the subjectively assessed probability of cancer recurrence, irrespective of any affective evaluation.

In this study, we used both instruments, the FoP-Q-12 and the CARQ-4, to answer several research questions. The objectives of this study were (a) to comparatively test the psychometric properties of the FoP-Q-12 (including the FoP-Q-5) and the CARQ-4, (b) to determine the degree of FoP in a large sample of cancer patients, (c) to examine the precise associations between FoP and general anxiety and health worries, and (d) to analyze the associations between sociodemographic and clinical factors and FoP.

## 2. Materials and Methods

### 2.1. Sample of Cancer Patients

In Germany, patients suffering from oncological diseases are generally offered the opportunity to participate in a rehabilitation program to regain physical and psychosocial functioning. During that program, the participants receive multiple treatments tailored to individual needs. A total of 2250 consecutive patients treated in a German cancer rehabilitation clinic were asked by the medical staff of the clinic to take part in this study between July 2022 and June 2023. Inclusion criteria were a confirmed cancer diagnosis, 18 years of age and above, and the absence of severe cognitive and/or verbal impairment that would interfere with the patient’s ability to give informed consent and to complete the questionnaires. Informed consent was obtained from the participants after they were given a full explanation of the purpose and nature of the data collection and storage. This study was approved by the Ethics Committee of the Medical Faculty of the University of Leipzig with the approval number: 513/21-ek.

### 2.2. Instruments

The following instruments were used in this study:

- **FoP-Q-12:** The Fear of Progression Questionnaire-12 (FoP-Q-12) [32] is a 12-item, short-form version of the original 43-item Fear of Progression Questionnaire (FoP-Q) [28]. The items are scored on a five-point Likert scale, ranging from 1 (‘never’) to 5 (’very often’). The sum score of the FoP-Q-12 ranges from 12 to 60. Scores of 34 or above indicate dysfunctional levels of FoP [33,34]. Recently, a 5-item short-form version of the FoP-Q-12 has been developed [30], called FoP-Q-RS. Here, we prefer the notation FoP-Q-5.
- **CARQ-4:** The CARQ-4 [31] consists of four questions concerning the fear of cancer recurrence. Three items were taken from the Fear of Cancer Recurrence questionnaire (FCR) [35]. One supplementary item was designed to assess the perceived risk of cancer recurrence. The first three items had to be answered using an 11-point Likert scale, ranging from 0 to 10. Item 4 asks for the subjectively assumed probability of cancer recurrence (scores between 0 and 100), and this estimated probability is also transformed to a 0–10 range. Therefore, the total score is in a range from 0 to 40. Scores of 12 and above indicate heightened levels of FoP.
- **GAD-7:** The Generalized Anxiety Disorder Questionnaire-7 (GAD-7) is a one-dimensional instrument designed to detect symptoms of generalized anxiety disorder as it was defined in the DSM-IV [36]. For each of the seven items, there are four possible answer options: not at all (0), for several days (1), for more than half of the days (2), and nearly every day (3), resulting in a sum score range from 0 to 21.
- **WI-7:** The Whiteley Index-7 (WI-7) was designed to measure health anxiety/illness worry [37]. It is a short form of the original 14-item WI-14 [38]. There are five response options (0–4); the sum score ranges from 0 to 28.
- **PHQ-9:** The PHQ-9 [39] is a 9-item screening instrument for measuring depression. As with the GAD-7, there are four possible answers for each item, ranging from 0 (not at all) to 3 (nearly every day), which results in a sum score range from 0 to 27.

### 2.3. Statistical Analyses

The items of the FoP-Q-12 and CARQ-4 were tested with part-whole corrected Pearson correlations between item and scale value. Internal consistency was determined with Cronbach’s α coefficient. The impact of sex and age on FoP was tested with 2-way ANOVAs. Effect sizes *d* according to Cohen were calculated to describe group differences; in the case of more than two groups, we used the corrected *r*² coefficient. Missing values were estimated using expectation maximization [40]. All statistical analyses were calculated with SPSS version 27.

## 3. Results

### 3.1. Sample Characteristics

Of the 2250 eligible cancer patients, 1733 patients (response rate 77%) agreed to participate and to complete the questionnaires. Of these, 1545 (89%) answered all items of the questionnaires completely. To avoid a reduction in the sample size of more than 10% due to listwise exclusion, the total of 422 missing values (0.6% of all values) were replaced with the estimation algorithm. Of the total 1733 participants, 59.5% were women, and the mean age was M = 56.0 years (SD = 14.5 years). Further characteristics of the sample are presented in Table 1.

### 3.2. Mean Scores and Psychometric Properties of the FoP-Q-12 and CARQ-4

Item and scale mean scores of the FoP-Q-12 and the CARQ-4 are provided in Table 2. The FoP-Q-12 items with the highest degrees of affirmation were item 2 (being nervous prior to doctor’s appointments or periodic examinations), item 5 (having physical sensations, e.g., rapid heartbeat, stomachache, nervousness), and item 11 (worrying about what will become of the family). Out of the CARQ-4 items, item 1 (worried about the possibility of cancer recurrence) and item 3 (emotionally upset or distressed about possible cancer recurrence) showed the highest mean values.

The total mean score of the FoP-Q-12 and the CARQ-4 were 30.0 and 16.1, respectively. Given the cutoff ≥ 34 for the FoP-Q-12, 16.8% of the male and 47.1 of the female patients reported heightened FoP; the total prevalence was 34.9%. In the case of the CARQ-4, however, the corresponding prevalence rates were much higher: 46.2% for males, 68.2% for females, and 59.3% for the total sample, using the cutoff ≥12 for the CARQ-4 as recommended by the test authors [31].

All items contributed positively and substantially to the total scale scores, with r_it_ coefficients ranging from 0.51 to 0.73 (FoP-Q-12) and from 0.61 to 0.90 (CARQ-4); the Cronbach α coefficients were 0.895 and 0.915, respectively; see Table 2.

The right part of Table 2 shows the correlations with other questionnaires. The correlation between the two FoP questionnaires, FoP-Q-12 and CARQ-4, was *r* = 0.72. Regarding the three additional questionnaires, the correlations with the Whiteley Index were highest (0.77 and 0.74), followed by the GAD-7 (0.69 and 0.60) and the PHQ-9 (0.65 and 0.54).

Out of the items of the CARQ-4, item 4 (likelihood of cancer recurrence) showed markedly lower correlations with the other questionnaires (*r* between 0.39 and 0.53) than the first three items (*r* between 0.48 and 0.71).

The short form of the FoP-Q-12, the FoP-Q-5, reached an α coefficient of 0.77, and the correlations with the other questionnaires were similar to those of the FoP-Q-12.

### 3.3. Impact of Sociodemographic and Clinical Variables on FoP

**Figure 1:**
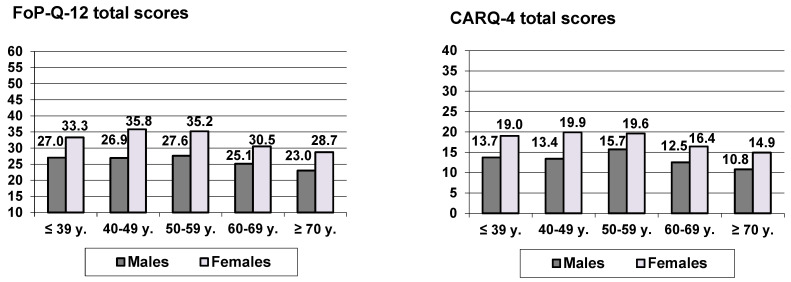
FoP-Q-12 and CARQ-4 mean scores, broken down by sex and age group.

Figure 1 presents sex and age differences in FoP scores. Both questionnaires show a very similar pattern. Females reported higher FoP levels than males, and there was a nonlinear relationship between age and FoP with an increase from the youngest age group to about 50 years and a decrease thereafter. The ANOVA results for the FoP-Q-12 and the CARQ-4 were as follows: FoP-Q-12: sex (F = 158.5, *p* < 0.001), age group (F = 19.4, *p* < 0.001), and sex * age group (F = 1.5, *p* = 0.213); CARQ-4: sex (F = 63.9, *p* < 0.001), age group (F = 10.9, *p* < 0.001), and sex * age group (F = 0.62, *p* = 0.649).

When considering CARQ-4 item 4 (perceived likelihood of cancer recurrence) separately, the following sex and age group mean scores emerged: males: 2.8 (≤39 y.), 3.0 (40–49 y.), 3.5 (50–59 y.), 2.9 (60–69 y.), and 2.5 (≥70 y.); and females: 3.7 (≤39 y.), 4.4 (40–49 y.), 4.4 (50–59 y.), 4.0 (60–69 y.), and 3.4 (≥70 y.).

Table 3 shows the impact of sex, age group (<60 vs. ≥60 years), and clinical parameters on the FoP. Because of the special interest in the CARQ-4 item 4, which only asks for probability, this item was also analyzed separately. Males reported less FoP than females (FoP-Q-12: M = 25.3 vs. M = 33.3; CARQ-4: M = 12.8 vs. M = 18.4). The older age group suffered from less FoP than the younger one (FoP-Q-12: M = 26.5 vs. M = 33.0; CARQ-4: M = 13.4 vs. M = 18.3). Cancer types with high levels of FoP were breast cancer, cancer of the female genital organs, and melanoma. With the exception of surgery, all treatments (radiation, chemotherapy, hormone therapy, and antibody therapy) were associated with higher levels of FoP.

## 4. Discussion

The first aim of this study was to analyze the psychometric properties of the two FoP questionnaires FOP-Q-12 and CARQ-4, including their relationship to other FoP-related constructs. Both the FoP-Q-12 and the CARQ-4 reached excellent Cronbach’s α coefficients, with both above 0.90. All items contributed positively and significantly to the corresponding total scores.

The short form of the FoP-Q-12, the FoP-Q-5, showed a markedly lower α coefficient (α = 0.77), which is probably due to two reasons. First, the item number is lower than that of the FoP-Q-12, and second, the authors of the 5-item short-form version tried to match each of the five distinct dimensions of the FoP with a certain item instead of identifying five items with maximum similarity, which would have resulted in higher correlations between the items and, therefore, in a higher α coefficient.

Regarding the four items of the CARQ-4, Table 2 shows that the first three of the items are very similar, with part–whole-corrected item–total correlations above 0.80, while item 4, the estimated probability of cancer recurrence, has a certain relative independence. This may be due to the fact that this item does not ask about emotional aspects but instead asks for a rational assessment of probability. If a physician discovers a discrepancy between the patients’ estimation of the probability of cancer recurrence and probability resulting from the physician’s expertise, this provides him with a starting point for an in-depth explanatory discussion to give the patient a more realistic perspective.

The correlation between the two questionnaires FoP-Q-12 and CARQ-4, which claim to capture the same construct, was *r* = 0.72. This correlation was slightly higher than the correlations with the GAD-7 and the PHQ-9 (Table 3), but the associations with the Whiteley Index-7 were present in both cases (*r* = 0.77 and *r* = 0.74), even slightly stronger than the correlation between the two FoP questionnaires. These results confirm the associations between FoP, generalized anxiety, and (to a slightly lower degree) depression, which were often reported in the literature [41]. As a new insight, our results add that general health worries, as measured by the Whiteley Index-7, are even more strongly associated with FoP than generalized anxiety.

A promising tool for a deeper analysis of the common structure of FoP and related constructs, such as generalized anxiety or health worries, seems to be a network analysis approach. In a sample of hematological cancer survivors [42], the FoP-Q and the GAD-7 were administered, and the relationships between the 13 items of the FoP-Q’s subscale ‘affective reactions’ and the seven items of the GAD-7 were visualized in a network. This network structure illustrated the associations between the items of both questionnaires in addition to the common variance that was shared by all items.

The mean prevalence of FoP was about one third (34.9%) when measured with the FoP-Q-12 and was nearly doubled when considering the CARQ-4 (59.3%). This discrepancy illustrates a reason for the large range of FoP prevalence rates reported in the literature [5]. It would be helpful if several FoP questionnaires could be compared with a common metric. Such analyses have already been performed for depression [43], fatigue [44], and anxiety [45]. A common metric for the domain of FoP would help compare results of studies using different scales with different cutoffs. Unfortunately, thus far, such a common metric is missing.

Female participants reported markedly higher levels of FoP than male respondents, which is in line with other studies on FoP in cancer patients [3,19] and needs to be interpreted in a context of generally higher anxiety scores for women compared to those for men [46]. Regarding age, we observed a clear nonlinear trend: an FoP increase in ages from 18 to about 50 years old and, thereafter, a decrease. However, if we construct only two age groups (up to 60 years and over 60 years old), the comparison of these groups indicates a decrease with increasing age. This apparent contradiction is due to the fact that the age groups 18–39 years and 40–49 years old are smaller than the older age groups; therefore, the lower FoP scores of young cancer patients are less noticeable in a two-group comparison. Multiple studies have examined age differences in FoP, sometimes with non-significant results [23,47], sometimes with negative correlations—e.g., [15,21]—and sometimes with a positive correlation [22]. The non-linear relationship found in our study shows that the sign of the association between FoP and age depends on the age range of the sample of cancer patients. Among older patients, a negative sign is to be expected. Correlation coefficients are generally useful statistical tools to express the character of an association between two variables. In the context of FoP, correlations seem to be insufficient. Regarding FoP in the youngest age group, one should bear in mind that AYA (adolescent and young adult) cancer patients also suffer from multiple kinds of distress and worry [48].

The separate calculation of the sex and age differences for the CARQ-4 item 4 (perceived likelihood of cancer progression or recurrence) shows the same age pattern as that of the full scales. This means that the non-linear age dependency is not determined by the affective component of FoP; the cognitive assessment of the probability contributes to this curve in the same way.

For healthcare providers, this implies that women in the age range between 40 and 60 years old deserve special attention concerning their FoP. With regard to further research on FoP, our findings indicate that correlations do not sufficiently represent age dependencies.

Among the tumor types, breast cancer and cancer of the female genital organs were associated with the highest levels of FoP, while prostate cancer patients reported the lowest levels. Evidently, tumor site and sex are confounded in these cases, and it cannot be sufficiently clarified to what degree the relatively high FoP scores of females suffering from the two cancer types are due to their sex or the cancer itself. For a thorough clarification of the effects of sex and tumor sites, it would be necessary to consider tumors that occur in males and females with roughly equal magnitude, such as cancers of the digestive organs. There are studies that try to separate these factors [20]; however, our study was not designed to investigate these separate influential factors.

Concerning treatment, all options except surgery were associated with higher levels of FoP. While surgery seems to be an intervention with good chances for the successful treatment of many tumor types and relatively low worries about cancer recurrence, other treatments, such as radiation, chemotherapy, or antibody therapy, have more side effects and a more severe impact on patients’ physical and mental health, as well as a higher degree of patient worry about the future. However, it should also be noted here that the forms of treatment are confounded with factors such as the severity of the disease, the stage, and the threatening nature of the tumor. These assessments should therefore be interpreted with great caution; a causal effect of the treatment methods on the FoP cannot be inferred. Nevertheless, for healthcare providers, this signifies that all patients receiving treatment in addition to surgery may show an enhanced FoP.

It would be interesting to estimate the objective probability of cancer recurrence depending on the chosen treatment options and to compare these figures with the probability perceived by the patients as captured by question four of the CARQ-4; however, in the context of this study, we cannot provide comparison numbers, as they also depend on further characteristics of the tumors.

FoP is not only relevant for the cancer patients themselves. Family members or others caring for the patients also suffer from fear of progression with regard to their relatives’ cancer [33], and it is an interesting task for future research to relate the psychometric properties of the FoP questionnaires applied to the patients themselves with the properties of their relatives.

Several limitations of this study should be noted. The sample may not be representative of all cancer patients because it was restricted to those who attended a rehabilitation program. Patients with a very high cancer burden as well as those with a very low perceived burden may be underrepresented. As already mentioned, sex effects and effects of the tumor type are confounded and cannot be sufficiently disentangled. The same holds for the effects of tumor type and treatment. In the rehabilitation hospital, the clinical data concerning tumor stage, metastases, and curative vs. palliative treatment intention were only partly available. Therefore, we could not test the associations between these variables and FoP.

Though item four of the CARQ-4 provides us with one possibility of separating the cognitive estimation of fear of recurrence from its affective evaluation, the instrument is not sufficient for a thorough distinction between the cognitive and the affective aspect. The surprising result that the correlations between FoP and general health worries (as measured by the Whiteley Index) were even somewhat higher than the correlations of the two FoP instruments suggests that the FoP results may also depend on the specific questionnaire and that the results obtained in the literature depend, to a certain degree, on the chosen instrument.

## 5. Conclusions

The main conclusion of this study is that both the FoP-Q-12 and CARQ-4 are useful instruments for measuring FoP. The level of FoP is relatively high, and it strongly depends on the definition of cutoff scores. The prevalence of FoP was about one third (34.9%) when measured with the FoP-Q-12 and the corresponding cutoff and nearly 60% when using the CARQ-4. Further efforts are needed to determine appropriate cutoffs that enable a comparison between different FoP instruments. Patients receiving radiation therapy, chemotherapy, hormone therapy, or antibody therapy showed heightened levels of FoP. Even if we could not control for confounding factors in our analyses and, therefore, could not determine the specific reasons for these heightened levels of FoP, healthcare providers should be aware that these patients deserve special attention concerning their FoP.

## Figures and Tables

**Table 1:** Sociodemographic and clinical characteristics of the sample (*n* = 1733).

	*n*	%
Sex		
Males	702	40.5
Females	1031	59.5
Age group		
18–39 years	254	14.7
40–49 years	276	15.9
50–59 years	417	24.1
60–69 years	464	26.8
≥70 years	322	18.6
Occupational status ^(a)^		
Employed	996	57.7
Retired	589	34.1
Unemployed	63	3.7
Other	78	4.5
Education ^(a)^		
No formal qualification	13	0.8
Elementary school (8–9 years)	356	20.6
Junior high school (10 years)	527	30.5
High school/university (≥11 years)	830	48.1
Tumor site		
Breast	560	32.3
Prostate	309	17.8
Gastrointestinal tract	290	16.7
Hematological	202	11.7
Female genital organs	108	6.2
Urinary tract	87	5.0
Melanoma	49	2.8
Thyroid/endocrine glands	38	2.2
Male genital organs	29	1.7
Others	61	3.5
Treatment		
Surgery ^(a)^		
No	177	10.2
Yes	1556	89.8
Radiation therapy ^(a)^		
No	952	55.0
Yes	779	45.0
Chemotherapy ^(a)^		
No	882	51.1
Yes	843	48.9
Hormone therapy ^(a)^		
No	1247	72.5
Yes	473	27.5
Antibody therapy ^(a)^		
No	1452	84.7
Yes	262	15.3

^(a)^ Missing data not reported.

**Table 2:** Mean scores and correlations of the FoP-Q-12 and the CARQ-4 on item level and scale level (*n* = 1733).

	M	SD	r_it_	Correlations				
				FoP-12	CARQ-4	GAD-7	WI-7	PHQ-9
**FoP-Q-12 (item range: 1–5)**								
(1) Being afraid of disease progression	2.6	1.1	0.71	0.76	0.76	0.62	0.71	0.53
(2) Being nervous prior to doctor’s appointments or periodic examinations	3.0	1.3	0.66	0.72	0.61	0.56	0.56	0.45
(3) Being afraid of pain	2.4	1.2	0.60	0.67	0.47	0.43	0.59	0.44
(4) Being afraid of becoming less productive at work	2.4	1.4	0.57	0.66	0.38	0.43	0.42	0.45
(5) Having physical sensations, e.g., rapid heartbeat, stomachache, nervousness	2.8	1.3	0.65	0.72	0.50	0.59	0.54	0.54
(6) Being afraid of the possibility that the children could contract cancer	2.5	1.4	0.51	0.61	0.44	0.38	0.42	0.35
(7) Being afraid of relying on strangers for activities of daily living	2.3	1.2	0.56	0.64	0.42	0.39	0.51	0.45
(8) Being afraid of no longer being able to pursue hobbies	2.3	1.2	0.57	0.65	0.44	0.40	0.53	0.44
(9) Being afraid of severe medical treatments in the course of the illness	2.5	1.2	0.73	0.78	0.65	0.49	0.63	0.46
(10) Worrying that medication could damage the body	2.5	1.3	0.58	0.66	0.42	0.38	0.50	0.41
(11) Worrying about what will become of the family	2.8	1.3	0.63	0.70	0.52	0.48	0.54	0.43
(12) Being afraid of not being able to work anymore	2.0	1.3	0.55	0.63	0.36	0.36	0.39	0.40
FoP-Q-12 total score	30.0	10.4	α = 0.895	1.00	0.72	0.67	0.77	0.65
FoP-Q-5 total score (5 items)	12.8	4.6	α = 0.772	0.95	0.70	0.69	0.74	0.66
**CARQ-4 (item range: 0–10)**								
(1) Worries about the possibility of cancer recurrence	4.6	3.1	0.90	0.67	0.94	0.57	0.70	0.48
(2) Intrusion of worry about cancer recurrence on thoughts and activities	3.5	2.9	0.85	0.69	0.92	0.61	0.71	0.54
(3) Emotionally upset or distressed about possible cancer recurrence	4.4	3.2	0.89	0.71	0.94	0.59	0.71	0.50
(4) Likelihood of cancer recurrence	3.6	2.9	0.61	0.50	0.76	0.39	0.53	0.41
CARQ-4 total score	16.1	10.8	α = 0.915	0.72	1.00	0.60	0.74	0.54

M: Mean; SD: Standard deviation; r_it_: part–whole corrected item-scale correlation.

**Table 3:** Sociodemographic and clinical factors predicting FoP (*n* = 1733).

	FoP-Q-12		CARQ-4		CARQ-4: Item 4 (Probability)	
	M	SD	M	SD	M	SD
Sex						
Males	25.3	9.0	12.8	10.1	2.9	2.7
Females	33.3	10.0	18.4	10.7	4.1	2.9
*d*	0.84		0.54		0.43	
*p*	<0.001		<0.001		<0.001	
Age group						
<60 years	33.0	10.4	18.3	10.8	4.0	2.9
≥ 60 years	26.5	9.1	13.4	10.2	3.2	2.8
*d*	−0.67		−0.47		−0.28	
*p*	<0.001		<0.001		<0.001	
Tumor class						
Breast	32.8	10.2	17.4	10.7	3.7	2.8
Prostate	23.3	7.9	11.0	9.4	2.5	2.5
Digestive organs	28.8	9.9	14.8	10.6	3.3	2.9
Hematological	31.3	10.0	18.0	10.7	4.1	3.1
Female genital organs	34.4	9.4	20.4	10.7	4.8	3.0
Urinary tract	29.9	10.9	15.8	10.3	3.5	3.0
Melanoma	32.7	10.6	21.7	10.2	5.0	2.7
corrected *r²*	0.121		0.070		0.053	
*F*	37.8		21.0		15.6	
*p*	<0.001		<0.001		<0.001	
Treatment						
Surgery						
No	30.2	10.1	17.9	10.8	4.2	3.2
Yes	30.0	10.4	15.9	10.8	3.5	2.9
*d*	−0.02		−0.19		−0.23	
*p*	0.419		0.022		0.002	
Radiation therapy						
No	28.9	10.4	15.2	10.9	4.4	2.9
Yes	31.3	10.1	17.1	10.6	3.8	2.9
*d*	0.35		0.21		0.21	
*p*	<0.001		<0.001		0.003	
Chemotherapy						
No	28.3	10.3	15.0	10.7	3.3	2.8
Yes	31.9	10.1	17.3	10.8	3.9	3.0
*d*	0.35		0.21		0.21	
*p*	<0.001		<0.001		<0.001	
Hormone therapy						
No	29.0	10.2	15.5	10.8	3.5	2.9
Yes	32.7	10.3	17.7	10.5	3.9	2.8
*d*	0.36		0.21		0.14	
*p*	<0.001		<0.001		0.014	
Antibody therapy						
No	29.3	10.2	15.4	10.6	3.4	2.8
Yes	33.8	10.3	19.9	11.3	4.8	3.1
*d*	0.44		0.41		0.47	
*p*	<0.001		<0.001		<0.001	

*d:* effect size; *F*: test statistic, *p*: significance level; CARQ-4 probability: item 4 of the CARQ-4.

## Data Availability

Raw data supporting the conclusions of this article will be made available by the corresponding author on reasonable request.

## References

[1] Fodor L.A., Todea D., Podina I.R. (2023). Core fear of cancer recurrence symptoms in cancer survivors: A network approach. Curr. Psychol..

[2] Dinkel A., Herschbach P. (2018). Fear of progression in cancer patients and survivors. Recent Results Cancer Res..

[3] Luigjes-Huizer Y.L., Tauber N.M., Humphris G., Kasparian N.A., Lam W.W.T., Lebel S., Simard S., Smith A.B., Zachariae R., Afiyanti Y. (2022). What is the prevalence of fear of cancer recurrence in cancer survivors and patients? A systematic review and individual participant data meta-analysis. Psycho-Oncology.

[4] Simard S., Savard J., Ivers H. (2010). Fear of cancer recurrence: Specific profiles and nature of intrusive thoughts. J. Cancer Surviv..

[5] Lebel S., Mutsaers B., Tomei C., Leclair C.S., Jones G., Petricone-Westwood D., Rutkowski N., Ta V., Trudel G., Laflamme S.Z. (2020). Health anxiety and illness-related fears across diverse chronic illnesses: A systematic review on conceptualization, measurement, prevalence, course, and correlates. PLoS ONE.

[6] Lebel S., Ozakinci G., Humphris G., Mutsaers B., Thewes B., Prins J., Dinkel A., Butow P. (2016). From normal response to clinical problem: Definition and clinical features of fear of cancer recurrence. Support. Care Cancer.

[7] Sarkar S., Scherwath A., Schirmer L., Schulz-Kindermann F., Neumann K., Kruse M., Dinkel A., Kunze S., Balck F., Kröger N. (2014). Fear of recurrence and its impact on quality of life in patients with hematological cancers in the course of allogeneic hematopoietic SCT. Bone Marrow Transplant..

[8] Tran T.X.M., Jung S.-Y., Lee E.-G., Cho H., Kim N.Y., Shim S., Kim H.Y., Kang D., Cho J., Lee E. (2022). Fear of cancer recurrence and its negative impact on health-related quality of life in long-term breast cancer survivors. Cancer Res. Treat..

[9] Hong S.J., Shin N.-M., Jung S. (2020). A predictive model of fear of cancer recurrence for patients undergoing chemotherapy. Support. Care Cancer.

[10] Llewellyn C.D., Weinman J., McGurk M., Humphris G. (2008). Can we predict which head and neck cancer survivors develop fears of recurrence?. J. Psychosom. Res..

[11] Waroquier P., France D., Darius R., Isabelle M. (2022). Psychological factors associated with clinical fear of cancer recurrence in breast cancer patients in the early survivorship period. Psycho-Oncology.

[12] Melchior H., Buscher C., Thorenz A., Grochocka A., Koch U., Watzke B. (2013). Self-efficacy and fear of cancer progression during the year following diagnosis of breast cancer. Psycho-Oncology.

[13] Koch L., Jansen L., Brenner H., Arndt V. (2013). Fear of recurrence and disease progression in long-term (≥5 years) cancer survivors-a systematic review of quantitative studies. Psycho-Oncology.

[14] Carver C.S., Smith R.G., Antoni M.H., Petronis V.M., Weiss S., Derhagopian R.P. (2005). Optimistic personality and psychosocial well-being during treatment predict psychosocial well-being among long-term survivors of breast cancer. Health Psychol..

[15] Crespi C.M., Ganz P.A., Petersen L., Castillo A., Caan B. (2008). Refinement and psychometric evaluation of the impact of cancer scale. J. Natl. Cancer Inst..

[16] Coutts-Bain D., Sharpe L., Pradhan P., Russell H., Heathcote L.C., Costa D. (2022). Are fear of cancer recurrence and fear of progression equivalent constructs?. Psycho-Oncology.

[17] Humphris G.M., Watson E., Sharpe M., Ozakinci G. (2018). Unidimensional scales for fears of cancer recurrence and their psychometric properties: The FCR4 and FCR7. Health Qual. Life Outcomes.

[18] Mehnert A., Koch U., Sundermann C., Dinkel A. (2013). Predictors of fear of recurrence in patients one year after cancer rehabilitation: A prospective study. Acta Oncol..

[19] Hinz A., Mehnert A., Ernst J., Herschbach P., Schulte T. (2015). Fear of progression in patients 6 months after cancer rehabilitation a validation study of the fear of progression questionnaire FoP-Q-12. Support. Care Cancer.

[20] Hinz A., Herzberg P.Y., Lordick F., Weis J., Faller H., Brähler E., Härter M., Wegscheider K., Geue K., Mehnert A. (2019). Age and gender differences in anxiety and depression in cancer patients compared with the general population. Eur. J. Cancer Care.

[21] Clayton M.F., Mishel M.H., Belyea M. (2006). Testing a model of symptoms, communication, uncertainty, and well-being, in older breast cancer survivors. Res. Nurs. Health.

[22] Kruger A., Leibbrand B., Barth J., Berger D., Lehmann C., Koch U., Mehnert A. (2009). Course of psychosocial distress and health-related quality of life in patients at different age groups during cancer rehabilitation [Verlauf der psychosozialen Belastung und gesundheitsbezogenen Lebensqualitaet bei Patienten verschiedener Altersgruppen in der onkologischen Rehabilitation]. Z. Psychosom. Med. Psychother..

[23] Skaali T., Fosså S.D., Bremnes R., Dahl O., Haaland C.F., Hauge E.R., Klepp O., Oldenburg J., Wist E., Dahl A.A. (2009). Fear of recurrence in long-term testicular cancer survivors. Psycho-Oncology.

[24] Carver C.S., Smith R.G., Petronis V.M., Antoni M.H. (2006). Quality of life among long-term survivors of breast cancer: Different types of antecedents predict different classes of outcomes. Psycho-Oncology.

[25] Thewes B., Butow P., Zachariae R., Christensen S., Simard S., Gotay C. (2012). Fear of cancer recurrence: A systematic literature review of self-report measures. Psycho-Oncology.

[26] Rudy L., Maheu C., Körner A., Lebel S., Gélinas C. (2020). The FCR-1: Initial validation of a single-item measure of fear of cancer recurrence. Psycho-Oncology.

[27] Smith A.B., Gao M., Tran M., Ftanou M., Jegathees S., Wu V., Jefford M., Lynch F., Dhillon H.M., Shaw J. (2023). Evaluation of the validity and screening performance of a revised single-item fear of cancer recurrence screening measure (FCR-1r). Psycho-Oncology.

[28] Herschbach P., Berg P., Dankert A., Duran G., Engst-Hastreiter U., Waadt S., Keller M., Ukat R., Henrich G. (2005). Fear of progression in chronic diseases—Psychometric properties of the Fear of Progression Questionnaire. J. Psychosom. Res..

[29] Mehnert A., Herschbach P., Berg P., Henrich G., Koch U. (2006). Fear of progression in breast cancer patients—Validation of the short form of the Fear of Progression Questionnaire (FoP-Q-SF) [Progredienzangst bei Brustkrebspatientinnen—Validierung der Kurzform des Progredienzangstfragebogens PA-F-KF]. Z. Psychosom. Med. Psychother..

[30] Youssef Y., Mehnert-Theuerkauf A., Götze H., Friedrich M., Esser P. (2021). Rapid screener for the assessment of fear of progression in cancer survivors: The Fear of progression-Questionnaire Rapid Screener. Eur. J. Cancer Care.

[31] Thewes B., Zachariae R., Christensen S., Nielsen T., Butow P. (2015). The Concerns About Recurrence Questionnaire: Validation of a brief measure of fear of cancer recurrence amongst Danish and Australian breast cancer survivors. J. Cancer Surviv..

[32] Mehnert A., Berg P., Henrich G., Herschbach P. (2009). Fear of cancer progression and cancer-related intrusive cognitions in breast cancer survivors. Psycho-Oncology.

[33] Zimmermann T., Herschbach P., Wessarges M., Heinrichs N. (2011). Fear of progression in partners of chronically ill patients. Behav. Med..

[34] Herschbach P., Berg P., Waadt S., Duran G., Engst-Hastreiter U., Henrich G., Book K., Dinkel A. (2010). Group psychotherapy of dysfunctional fear of progression in patients with chronic arthritis or cancer. Psychother. Psychosom..

[35] Easterling D.V., Leventhal H. (1989). Contribution of concrete cognition to emotion: Neutral symptoms as elicitors of worry about cancer. J. Appl. Psychol..

[36] Spitzer R.L., Kroenke K., Williams J.B.W., Lowe B. (2006). A brief measure for assessing generalized anxiety disorder—The GAD-7. Arch. Intern. Med..

[37] Carstensen T.B.W., Ørnbøl E., Fink P., Pedersen M.M., Jørgensen T., Dantoft T.M., Benros M.E., Frostholm L. (2020). Detection of illness worry in the general population: A specific item on illness rumination improves the Whiteley Index. J. Psychosom. Res..

[38] Fink P., Ewald H., Jensen J., Sørensen L., Engberg M., Holm M., Munk-Jørgensen P. (1999). Screening for somatization and hypochondriasis in primary care and neurological in-patients. J. Psychosom. Res..

[39] Kroenke K., Spitzer R.L., Williams J.B.W. (2001). The PHQ-9—Validity of a brief depression severity measure. J. Gen. Intern. Med..

[40] Dempster A.P., Laird N.M., Rubin D.B. (1977). Maximum likelihood from incomplete data via the EM algorithm. J. R. Stat. Soc. Ser. B (Methodol.).

[41] Podina I.R., Todea D., Fodor L.-A. (2023). Fear of cancer recurrence and mental health: A comprehensive meta-analysis. Psycho-Oncology.

[42] Göbel P., Kuba K., Götze H., Mehnert-Theuerkauf A., Spitzer C., Hartung T., Esser P. (2023). Interconnectivity of fear of progression and generalized anxiety—Network analysis among a sample of hematological cancer survivors. Support. Care Cancer.

[43] Wahl I., Löwe B., Bjorner J.B., Fischer F., Langs G., Voderholzer U., Aita S.A., Bergemann N., Brahler E., Rose M. (2014). Standardization of depression measurement: A common metric was developed for 11 self-report depression measures. J. Clin. Epidemiol..

[44] Friedrich M., Hinz A., Kuhnt S., Schulte T., Rose M., Fischer F. (2019). Measuring fatigue in cancer patients: A common metric for six fatigue instruments. Qual. Life Res..

[45] Clover K., Lambert S.D., Oldmeadow C., Britton B., King M.T., Mitchell A.J., Carter G.L. (2022). Apples to apples? Comparison of the measurement properties of hospital anxiety and depression-anxiety subscale (HADS-A), depression, anxiety and stress scale-anxiety subscale (DASS-A), and generalised anxiety disorder (GAD-7) scale in an oncology setting using Rasch analysis and diagnostic accuracy statistics. Curr. Psychol..

[46] Löwe B., Decker O., Müller S., Brähler E., Schellberg D., Herzog W., Herzberg P.Y. (2008). Validation and standardization of the generalized anxiety disorder screener (GAD-7) in the general population. Med. Care.

[47] Deimling G.T., Bowman K.F., Sterns S., Wagner L.J., Kahana B. (2006). Cancer-related health worries and psychological distress among older adult, long-term cancer survivors. Psycho-Oncology.

[48] Yang Y., Li W., Wen Y., Wang H., Sun H., Liang W., Zhang B., Humphris G. (2019). Fear of cancer recurrence in adolescent and young adult cancer survivors: A systematic review of the literature. Psycho-Oncology.

